# Platypnea-Orthodeoxia Syndrome After the Bentall Procedure

**DOI:** 10.7759/cureus.64645

**Published:** 2024-07-16

**Authors:** Yusuke Setonaga, Hiromasa Hayama, Hidehiko Hara, Yoshiyuki Yazaki, Masato Nakamura

**Affiliations:** 1 Division of Cardiovascular Medicine, Toho University Ohashi Medical Center, Tokyo, JPN

**Keywords:** cardiology devices, patent forman ovale (pfo), percutaneous pfo closure, bentall procedure, platypnea-orthodeoxia syndrome, pos

## Abstract

Platypnea-orthodoxia syndrome (POS) is a clinical condition that causes dyspnea and hypoxia in the sitting and standing positions. In this case, a 67-year-old man showed hypoxemia after undergoing the Bentall procedure that worsened in the standing position during rehabilitation. Contrast-enhanced computed tomography of the thorax and abdomen revealed no cause of respiratory failure. POS was suspected as the cause of the positional exacerbation of oxygen saturation. A bubble study showed a positive grade IV within three heartbeats on transthoracic echocardiography, which also confirmed an intracardiac shunt caused by a patent foramen ovale (PFO). Percutaneous PFO closure was performed, and hypoxemia was immediately resolved. Various factors were considered to cause the POS, including right heart failure, constrictive pericarditis, and postoperative adhesions, and each of these factors was discussed. POS after open-heart surgery is very rare. This is the first reported case of POS treated with a closure device following the Bentall procedure.

## Introduction

Platypnea means flat respiration, and orthodeoxia indicates hypoxemia exacerbated by standing or sitting. The cause of platypnea-orthodeoxia syndrome (POS) varies from case to case and is thought to be a combination of anatomical and functional factors [1]. Although several etiologies exist, the pathophysiology of how endocardial abnormalities cause POS is not fully understood. Because positional dyspnea is not present at birth and is developed later in life, acquired factors, in addition to endocardial abnormalities, are necessary for the development of POS. These anatomical factors include intracardiac and intrapulmonary shunts, as well as pulmonary ventilation imbalance [2]. Functional factors include age-related spinal deformity, enlargement and extension of the aorta, and clockwise rotation of the heart [3]. Since the first report of this rare condition by Burchell et al. in 1949, the number of case reports has been increasing, but the condition has not gained extensive recognition [4-7]. There are a few reports of POS after open-heart surgery and associated successful shunt closure with percutaneous devices [8,9]. We report a case of POS after the Bentall procedure that was successfully treated using a percutaneous device.

## Case presentation

**Figure 1:**
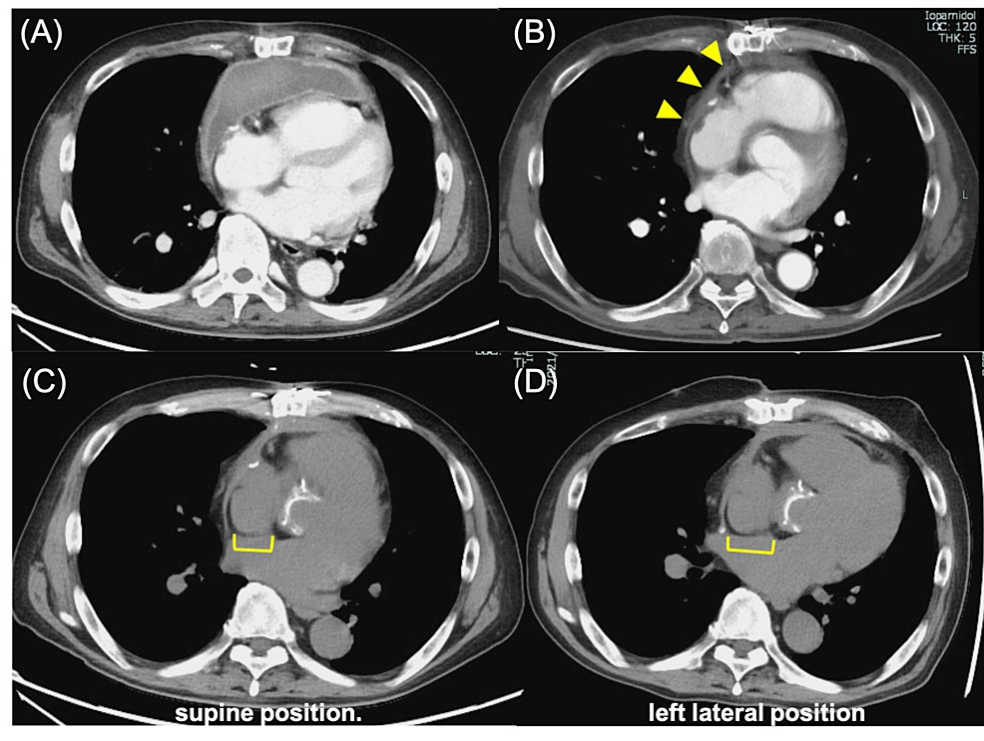
Chest computed tomography after the Bentall procedure. (A) A few days after the Bentall procedure, a pericardial hematoma was observed. (B) After drainage of the pericardial hematoma, pleural thickening was observed as indicated by the yellow arrow. (C) Image taken before patent foramen ovale closure in the supine position. (D) Image taken with the patient in the left lateral position. The atrial septum was stretched more than in the supine position.

**Figure 2:**
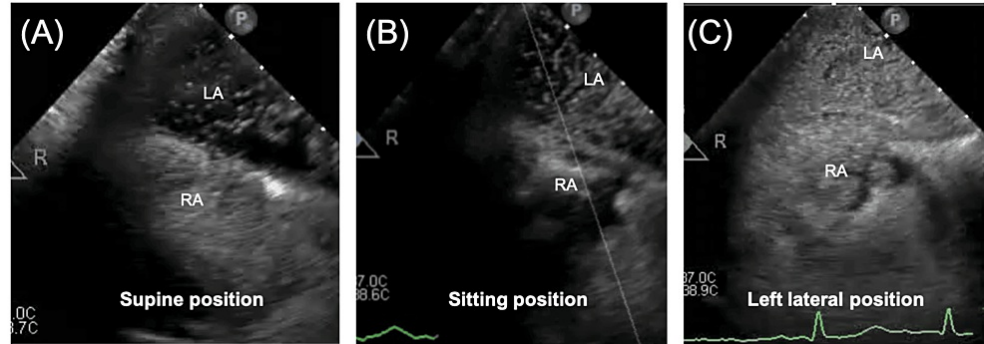
Transesophageal echocardiography maximal bubble study images for each positional change and lung perfusion scintigraphy. (A) Grade IV bubble inflow into the left atrium was observed. (B) The inflow of bubbles was increased compared to the supine position. (C) The entire left atrium was filled with bubbles, and more bubbles were observed than in other positions.

**Figure 3:**
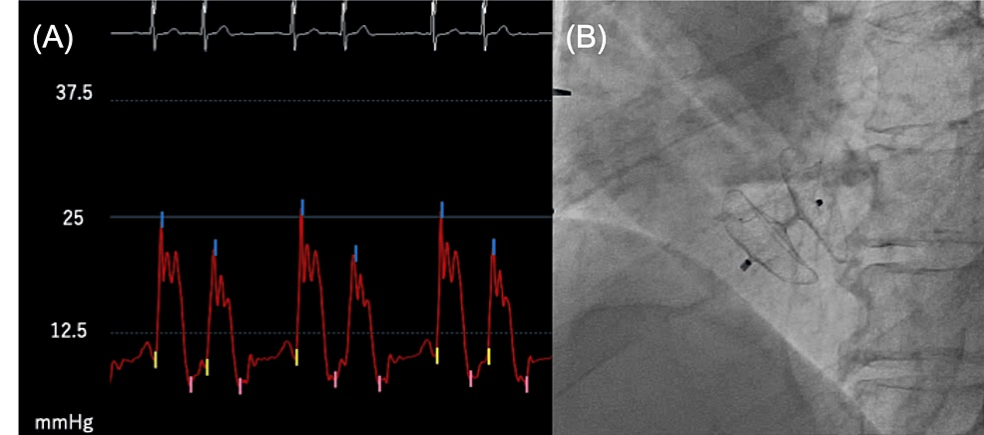
Right ventricular pressure before the patent foramen ovale (PFO) closure and after the PFO closure device implanted. (A) Right ventricular pressure was 27/7/9 mmHg. A dip and plateau were observed. (B) AMPLATZER Cribriform Device (30 mm, St. Jude Medical, Inc.) was implanted without complications.

A 67-year-old man presented with acute chest pain and dyspnea. He previously underwent an initial Bentall procedure for aortic valve annulus dilatation 17 years ago. Bentall procedure is the gold standard in the treatment of patients requiring aortic root replacement [10,11]. On this occasion, he suddenly developed chest pain and dyspnea and was transported to the hospital for emergency treatment. He was diagnosed with acute aortic regurgitation due to cusp rupture of the aortic bioprosthetic valve and underwent an urgent second Bentall procedure. One week after surgery, a pericardial hematoma was diagnosed (Figure 1A), which was improved by pericardial drainage; however, pleural thickening remained (Figure 1B). Subsequently, the patient began to exhibit shortness of breath during rehabilitation and positional changes with associated decreased oxygen saturation levels. At that time, a decrease in oxygen saturation of more than 5% was observed in the sitting and left lateral recumbent positions, which is a finding associated with POS. Blood tests showed a mildly elevated N-terminal pro-b-type natriuretic peptide (NT-proBNP) of 397 pg/mL. No other abnormal findings were found on the blood counts, liver and renal functions, or electrolyte tests. Chest radiographs showed an enlarged right pulmonary artery with a cardiothoracic ratio of 45%. An electrocardiogram showed supraventricular extrasystoles, and a transthoracic echocardiogram revealed a mildly decreased left ventricular ejection fraction of 50% (Modified Simpson) and an enlarged left atrial diameter of 50 mm. Right ventricular function was impaired in both contractility and diastolic function, as indicated by a tricuspid annular plane systolic excursion of 9 cm, a right ventricular fractional area change of 28%, an S' velocity of 9 cm/s, a tricuspid valve (TV) E/A ratio of 1.96, and a TV E wave deceleration time of 129 ms. An echocardiogram with bubble contrast and intravenous agitated saline showed a grade IV right to left shunt. Pulmonary blood flow scintigram showed accumulation in extrapulmonary organs, such as the brain and kidneys, and the right-left shunt rate was 18.2%. Based on these findings, a transesophageal echocardiography was performed to search for shunting disease, which could be the cause of POS, and a patent foramen ovale (PFO) was observed. An echocardiogram with bubble contrast performed at that time showed that the inflow of bubbles worsened in the sitting and left lateral recumbent positions (Videos 1-3 and Figures 2A-2C). As the worsening of the right-left shunt with positional change was already confirmed, a chest computed tomography (CT) scan was taken in the left lateral recumbent position and compared with that in the supine position. The atrial septum was stretched in the left lateral recumbent position, presumably due to the postoperative adhesion of the aorta and the right atrium, which was thought to further exacerbate the right-left shunt (Figures 1C-1D). Chest CT showed no obvious stretching of the aorta or compression of the right atrium, but pleural thickening was observed, mainly in the anterior aspect of the right ventricular system (Figure 1B). Cardiac catheterizations showed mean right and left atrial pressures of 11 and 7 mmHg, respectively, indicating an increase in the mean right atrial pressure. Furthermore, a dip and plateau were observed in the right ventricular pressure, and contractile pericarditis was suspected (Figure 3A). These findings suggest that the POS in this patient was caused by a combination of the primary factor, PFO, and secondary factors, including atrial septal extension due to positional changes, right heart failure, and constrictive pericarditis. The patient had undergone two open-heart surgeries and was considered at considerable risk for another. Percutaneous PFO closure was performed under intracardiac echocardiographic guidance. The balloon sizing was 18 mm, and closure was performed with a 30 mm AMPLATZER Cribriform Device (St. Jude Medical, Inc., Saint Paul, MN) (Figure 3B). Transthoracic echocardiography the day after the procedure showed a marked improvement in the right-left shunt, and oxygen saturation levels of the air remained above 96% in the room regardless of position. 

**Video 1:** Transesophageal echocardiography maximal bubble study video in the spine position. Grade IV bubble inflow into the left atrium was observed.

**Video 2:** Transesophageal echocardiography maximal bubble study video in the sitting position. The inflow of bubbles was increased compared to the supine position.

**Video 3:** Transesophageal echocardiography maximal bubble study video in the left lateral position. The entire left atrium was filled with bubbles, and more bubbles were observed than in other positions.

## Discussion

POS was first reported by Burchell in 1949 [4]. The causes of POS vary widely from case to case and include intracardiac shunts, extracardiac shunts, and pulmonary parenchymal disease, with 90% of cases being caused by intracardiac shunts. Primary causes of intracardiac shunts include atrial septal defects, atrial septal, and PFO [1,2]. Secondary factors such as anatomical aortic changes and pneumonectomy are also thought to contribute to the onset of POS [2,3]. The first step in diagnosing an intracardiac shunt is to perform transthoracic echocardiography with a bubble/contrast study, and if a bubble is seen flowing into the left ventricular system within three heartbeats, the presence of an intracardiac shunt is suspected [2]. Following this, a transesophageal echocardiogram is performed to search for detailed morphological abnormalities. If an abnormality still cannot be detected, cardiac magnetic resonance imaging should be considered. Surgery was previously the first choice for treatment, but more recently, percutaneous transcatheter closure is often the treatment of choice due to its minimal invasiveness and cost-effectiveness. Only limited reports on the occurrence of POS after open-heart surgery have been published. In this case, pericardial thickening remained after postoperative drainage of pericardial hematoma, leading to the pathogenesis of constrictive pericarditis, which may have influenced the development of POS. The frequency of constrictive pericarditis after open-heart surgery has been reported to be 0.2%-0.3% [12]. Pericardiectomy is the primary treatment for constrictive pericarditis. In addition, the reduction of the right ventricular systolic function after open-heart surgery may have also contributed to the increased right atrial pressure. Mattei et al. reported that the causes of right ventricular dysfunction after open-heart surgery include volume overload, pressure overload, myocardial ischemia, inflammation, oxidative stress, pulmonary embolism, pericardial effusion, and arrhythmias [13]. In this case, increased right atrial pressure due to right ventricular dysfunction and postoperative adhesions between the aorta and the right atrium caused stretching of the atrial septum upon changing positions, which triggered the development of POS. Right ventricular function improved three months after closure, with a tricuspid annular plane systolic excursion of 17 cm, right ventricular fractional area change of 42%, and S' velocity of 9.7 cm/s. This improvement was attributed, in part, to the fact that the patient obtained sinus rhythm after the closure. In conclusion, we report a case of POS, resulting from various factors, including constrictive pericarditis following the Bentall procedure, which was successfully managed by closing the PFO with a percutaneous approach.

## Conclusions

POS after open-heart surgery is very rare. This is the first reported case of POS treated with a closure device after the Bentall procedure. POS should be considered as a cause of respiratory failure after cardiovascular surgery.
